# Comments on Kroken et al.'s "Long-term frailty trajectory in older ICU survivors requiring mechanical ventilation: a prospective cohort study in Norway"- Author's reply

**DOI:** 10.1016/j.aicoj.2026.100186

**Published:** 2026-10-14

**Authors:** Shirin Kordasti Frisvold, Bjorn Anders Kroken, Marina Espinasse, Hans Flaatten

**Affiliations:** The Arctic University of Norway, Norway

We thank You et al. for their careful reading of our study.

We agree that survivor-based percentages may overstate recovery, as the decline in frailty between three and 12 months may partly reflect deaths among the frailest patients. We chose survivor-based percentages to describe variation in CFS among those alive. Counts for the full cohort shown in Figure 2 [1]. State occupancy for the inception cohort is:

**Table:** 

	3 months	12 months
Robust (CFS 1–3)	58 (16.8 %)	67 (19.4 %)
Prefrail (CFS 4)	35 (10.1 %)	27 (7.8 %)
Frail (CFS 5–9)	81 (23.4 %)	59 (17.1 %)
Dead	161 (46.5 %)	180 (52.0 %)
Withdrawn consent	11 (3.2 %)	13 (3.8 %)
Alive, non-frail (CFS 1–4)	93 (26.9 %)	94 (27.2 %)

At cohort level, the proportion alive and frail fell from 23% to 17%. The proportion alive without frailty remained stable at 27 %. Equal numbers of frail patients left the frail group through death (n = 15) and through improvement (n = 15) (Supplementary Table S3b) [1]. Therefore, both depletion and recovery contributed. Also, the number of robust patients increased from 58 to 67. We agree that future studies should report death as a separate outcome state.

The aim of the regression was exploratory: to describe factors across the study course associated with CFS change, rather than estimating ICU treatment effects. We agree that institutionalisation at 12 months may act as a collider [2]. Without this covariate the associations were: ICU length of stay (β 0.38 per SD, 95% CI 0.03 to 0.73), rehabilitation within 12 months (β 0.67, 0.39 to 0.95), hospital readmission (β 0.44, 0.15 to 0.72), RRT (β −0.58, −0.89 to −0.26). In a baseline-only model, none of the ICU exposures were significantly associated with CFS change.

We acknowledge that informant and assessment mode differed between baseline (mainly proxy and in person) and follow-up (patient or proxy, by mail and telephone), and that this could affect CFS scores. However, at all time points, CFS rated habitual function rather than day-to-day status. Pictograms were used at every assessment, and the three- and 12-month assessments used the same mode. As patient and proxy ratings were not obtained for the same patients, we cannot separate informant effects, although previous research shows small inter-rater variability [3].

We noted the association between frailty and central nervous system disease in the Discussion but did not perform a sensitivity analysis. Excluding these patients attenuated the mean CFS increase among survivors from 1.49 (95% CI 1.20–1.78) to 0.94 (95% CI 0.70–1.19) at three months and from 1.18 (95% CI 0.89–1.46) to 0.70 (95% CI 0.45–0.95) at 12 months. The increase remained statistically significant (p < 0.001). A detailed subgroup analysis will follow. Results from the full cohort should be interpreted with case-mix in mind.

We share the aim of translating these trajectories into early counselling and support future research in this area.

Sincerely, Shirin K Frisvold,MD, PhD Bjørn Anders Kroken, MD, Marina Espinasse, PhD, and Hans Flaatten MD, PhD

## Authors' contributions

SKF and BAK analysed the data. ME supervised the statistical analysis. SKF wrote the original draft, and all authors edited and approved the final draft.

## Declaration of Generative AI and AI-assisted technologies in the writing process

The authors used Claude (Anthropic) for assistance with R codes for the additional analysis and language editing. The authors reviewed and edited the content as needed and take full responsibility for the content of the publication.

## Ethics approval and consent to participate

The study was approved by the Norwegian Regional Committee for Medical and Health Research Ethics, (172,784/REK North). Written informed consent was obtained from the patient or proxy in the acute phase or at follow up. If a patient died in the acute phase before consenting, inclusion was done by deferred consent, according to local regulations.

## Funding

This study was funded by the Northern Norway Regional Health Authority (181021).

## Availability of data and material

The datasets used and/or analysed during the current study are available from the corresponding author on reasonable request.

## Declaration of competing interest

The authors declare that they have no known competing financial interests
